# Antarctic fungi: a bio-source alternative to produce polyunsaturated fatty acids (PUFAs)

**DOI:** 10.1128/spectrum.01372-25

**Published:** 2026-01-13

**Authors:** Patrizia De Rossi, Alfredo Ambrico, Antonella Del Fiore, Mario Trupo, Luciano Blasi, Marzia Beccaccioli, Luigi Faino, Andrea Ceci, Oriana Maggi, Anna Maria Persiani, Massimo Reverberi

**Affiliations:** 1 Italian National Agency for New Technologies, Energy and Sustainable Economic Development, ENEA Casaccia Research Centre, Rome, Italy; 2 Italian National Agency for New Technologies, Energy and Sustainable Economic Development, ENEA Trisaia Research Centre 97026, Rotondella, Italy; 3 Department of Environmental Biology, Sapienza University of Rome, Rome, Italy; Institute of Microbiology, Chinese Academy of Sciences, Beijing, China

**Keywords:** microfungi, biomass, PUFAs, Antarctic fungi, linolenic fatty acid, bio-sources

## Abstract

The Antarctic ecosystems are a combination of conditions, including extremely low values of temperature and humidity. Nonetheless, some organisms, such as fungi, can adapt to these extreme conditions. The environmental temperature is one of the parameters thoroughly affecting the structure and composition of fungal membrane lipids. The psychrophilic fungi generally increase the disorder within macromolecules to maintain membrane fluidity at low temperatures. To do so, Antarctic fungi increase the proportion of unsaturated fatty acids that allow maintaining a semi-fluid state of the membranes and survive at extremely low temperatures. This ecological feature may be of interest for the characterization of phenotypical traits of the ecological adaptation of these fungi to the extreme environmental conditions of Antarctica. Moreover, this can be of inspiration to find solutions inspired by nature for alternative sources of polyunsaturated fatty acids (PUFAs) for diets of humans and animals. We characterized three fungal strains isolated from Antarctica and set up a laboratory/small-scale production of fungal biomass with a high content of beneficial PUFAs. In detail, three fungal species previously isolated from Antarctic environmental matrices were tested and identified at the genome level. We also conducted growth experiments to determine the effects of temperature and substrate on biomass and PUFA production. The results showed that these fungi have a high percentage of PUFAs compared to saturated ones; the growth at low temperatures (10°C) increases the production of linolenic acid (C18:3) while the biomass amount (yield) depends on the composition of the growth substrate; a satisfying qualitative-quantitative production is achieved using agri-food chain waste products, such as brewing and whey waste, as a growth substrate.

**IMPORTANCE:**

The presence of polyunsaturated fatty acids (PUFAs) in the diets of humans and animals is gaining attention because PUFAs have several recognized functional properties: they modulate immune response, have anti-allergic and anti-inflammatory activity, have a cardio-protective effect, and reduce blood LDL cholesterol levels. Since few foods naturally contain high levels of PUFAs, human diets are often deficient in these fatty acids, which is why supplementation is often needed. Regarding this, it is necessary to develop efficient industrial processes capable of producing good-quality PUFAs and in quantity, even using agri-food chain waste products as carbon and nitrogen sources (in our case, spent yeast from brewing and whey waste). Like microorganisms, we took into consideration Antarctic fungi because they can adapt to very low temperatures also by increasing the proportion of unsaturated fatty acids that allows maintaining a semi-fluid state of their membranes. The innovation of our study consists of a characterization of phenotypical traits on PUFA production by fungal strains from Antarctica in different cultural conditions, including the use of agri-food processing wastes. The combination of these conditions can be an inspiration for a new, alternative, and sustainable way to produce PUFAs with extreme microorganisms.

## INTRODUCTION

In psychrophilic organisms, there is a direct correlation between the adaptation to low temperatures and the degree of unsaturation of membrane lipids: the lower the temperature, the greater the amount and degree of unsaturation in the fatty acids incorporated into membrane lipids. Some microorganisms, such as fungi, possess a remarkable phenotypic plasticity (1) that allows them to adapt—also through epigenetic adaptive memory—to extreme environmental conditions (2–4). The Antarctic ecosystems are combinations of extreme conditions that include low temperature and humidity, poor availability of organic matter, and high salt stress conditions, among others. This leads to low biodiversity and low biomass production (5–7). Few organisms can adapt to such conditions; among these are microfungi that possess a high surface-area-to-volume ratio and exhibit significant phenotypic and metabolic plasticity. These traits are displayed in their communities, which, despite being simple in composition and structure, are physiologically complex. This complexity paves the way for them to face extreme environments (8).

The interest of this work focused particularly on the phenotypic fungal plasticity in terms of polyunsaturated fatty acid (PUFA) synthesis, as one of the main adaptive responses to low temperatures. PUFAs are found in every organism, but humans cannot synthesize some of them, such as linoleic acid, which is crucial for the synthesis of others (e.g., linolenic and arachidonic acid). The presence of PUFAs, ω3−ω6−ω9, in animal and human diets is gaining attention since PUFAs may especially act as regulators in the blood cholesterol level and are beneficial for the cardio-circulatory system (9). Among PUFAs, eicosapentaenoic acid (EPA; 20:5n-3) and docosahexaenoic acid (DHA; 22:6n-3), which are n-3 long-chain PUFAs widely referred to as ω-3 oils, were reported to prevent the development of obesity in rodents and humans (10). For this reason, dietary supplements containing these kinds of fatty acids are commercially available for health maintenance and the prevention of chronic diseases.

The commercial production of ω-3 oils, such as EPA and DHA, has primarily depended on marine fish oils to date, but the application of fish oils is often hampered by difficulties, including seasonal variations, marine pollution, and the high processing costs (11, 12). In addition, mass-scale fishing to match the increasing demand for fish oils is no longer sustainable (13). Psychrophilic fungi, modulating the saturated:unsaturated lipid ratio in response to temperature variations, have recently gained particular attention. They can produce amounts of PUFAs (14–16) such as γ-linolenic acid, EPA, and DHA, purposed as food and feed additives. In the search for an efficient and sustainable microbial PUFA production, microbial strains could provide a nature-based solution. Notably, fungi can provide goods and ecosystem services for humans and animals, including nutritional support. Based on the type of substrate used for their growth, fungi can be employed for different purposes (17). Fermentation techniques enable the controlled production of fungal biomass by allowing the manipulation of growth parameters like pH, dissolved oxygen, agitation, and nutrients. This control leads to a quantitative and qualitative increase in fungal growth and the enhanced production of commercially valuable metabolites (18). Indeed, in recent studies, it has also been shown that the composition of the substrate and the set of growth parameters strongly influence the production of PUFAs in microorganisms isolated from seawater and soil (19, 20). Furthermore, the control of the growth parameters allows standardizing the production of specific compounds and making them repeatable over time.

This study aims to characterize phenotypical traits in the production of PUFAs by microfungi isolated from Antarctica under different cultural conditions in a context of circular economy.

## MATERIALS AND METHODS

### Fungal strains

The following strains were used: *Cordyceps farinosa* (Holmsk.) Kepler, B. Shrestha and Spatafora (formerly *Paecilomyces farinosus* [Holmsk.] A.H.S. Br. and G. Sm.); C*adophora malorum* (Kidd and Beaumont) W. Gams, isolated from moss of Kay Island (74°05′S, 165°17′E); and a fungal strain of *Agonomycetales* (formerly *Mycelia sterilia*) (later identified as *Epicoccum nigrum*), isolated from soil under moss of Starr Nunatak (75°54′S, 162°35′E) during the 10th summer Italian expedition in Victoria Land, Antarctica, 1994–1995. The strains were obtained from the culture collection of the Fungi Biodiversity Laboratory (FBL) of the Department of Environmental Biology, Sapienza University of Rome, where they are preserved with the labels FBL 167, FBL 175, and FBL 181, respectively.

### Genetic characterization of fungal species

*P. farinosus* FBL 167, *C. malorum* FBL 175, and *E. nigrum* FBL 181 were grown in Potato Dextrose Broth (PDB). After 7 days, the mycelium was harvested by filtering through Whatman qualitative filter paper grade 1, washed with sterile distilled water, and lyophilized. The mycelium was ground in the presence of liquid nitrogen, and 30 mg was used to extract the DNA following the 3 CTAB method (21). Total extracted DNA was visualized by gel electrophoresis. Quantity and quality checks were evaluated by spectrophotometer (Nanodrop, Thermo Scientific). One hundred nanograms of pure DNA was amplified by PCR with fungal ITS universal primers. The pair of universal primers ITS1 (5′-TCCGTAGGTGAACCTGCGG-3′) and ITS4 (5′-TCCTCCGCTTATTGATATGC-3′) amplified the rDNA (22). Amplification was performed with BIOTAQ DNA Polymerase (Bioline, Meridian Bioscience) following the protocol reported by the manufacturer. Identification of fungal species was performed by sequencing the purified amplicon with the ISOLATE II PCR and Gel Kit (Bioline, Meridian Bioscience) using the Sanger sequencing approach. To confirm the barcode identification, the whole genome of the three isolates was sequenced using nanopore sequencing (MinION) performed following the manufacturer’s indications. DNA extraction for nanopore sequencing was performed as described in Seidl et al. (23). The DNA library was constructed using the SQK-LSK109 and EXP-NBD104 kit and run on an R9.4.1 flow cell system.

DNA sequencing was performed using MinION and assemblies were generated using HiFiasm software.

### Selection of optimal fungal growth conditions in shaken flasks

Preliminary tests were carried out to evaluate the highest biomass yield of the three fungi grown on different liquid media (Table S1). In the first experiment, 25 mL of sterilized liquid medium in 100 mL flasks was inoculated with 100 µL of FBL 167, FBL 175, or FBL 181 spore suspension (10^4^ CFUs/mL) and incubated in an orbital shaker (Comecta Mod. WY-200) at 25°C and 180 rpm. To prepare the inoculum, mycelium and conidia were scraped from fresh fungal cultures grown on PDA at 25°C for 8–10 days and suspended in sterile water. This experiment allowed us to select three substrates (S10, S11, and S15) based on their fungal biomass yields. These selected substrates were used in a second experiment with the same fungi at different temperatures. Specifically, one set of flasks, consisting of two replicates for each fungus and for each substrate, was incubated at 10°C for 10 days, and one set of flasks was incubated at 25°C for 10 days. In another set, the temperature was maintained at 25°C for the first 6 days and then lowered to 10°C for the last 4 days (hereinafter referred to as 25°C and 10°C). For all experiments, after incubation times, the culture liquid was centrifuged at 13,000 *g* for 10 min at 4°C, and the pellet was washed two times with sterile water. Subsequently, the fungal biomass was freeze-dried using a pilot lyophilizer (Christ, Loc-1M). The dried fungal biomass was weighed and stored until it was used for chemical analysis. All experiments were repeated two times.

### Production of fungal biomass in the bioreactor

The experiments were conducted in a 5 L bioreactor (BIOSTAT B, Braun Biotech International) using the media S10, S11, and S15. The fungal culture was carried out in batch mode with a working volume of 3 L. For each fungal species, a 96-hour-old preculture in PDB was used as a starter at 5% (vol/vol). During the fermentation process, the temperature of the liquid culture was first maintained at 25°C for 5 days and then lowered to 10°C for the other 3 days. Aeration and agitation rates were controlled at 2 L/min and 200 rpm, respectively. The pH was set at 7 and controlled by adding sterile solutions of 2 M sodium hydroxide and 5% sulfuric acid. At the end of the culturing process, the mycelium was recovered by centrifugation at 13,000 *g* for 15 min at 4°C, washed with sterile water, and freeze-dried. The dried biomass was gravimetrically determined and stored in vacuum packaging until the subsequent lipid analysis. All experiments were repeated three times.

### Fatty acid profiling in fungi by gas chromatography (GC) analysis

Fatty acids were extracted from mycelium grown in shaken flasks and in the bioreactor using S10, S11, and S15 media at 10°C for 10 days, at 25°C for 10 days, and at 25°C and 10°C. Fatty acids were derivatized into fatty acid methyl esters (FAME) following a hydrolysis- and methylation-based procedure. To 200 mg of lyophilized mycelium ground in liquid nitrogen were added the nonadecanoic acid internal reference standard for (semi)quantitative analysis and 2 mL of 0.5 M KOH in methanol; this solution was then maintained at 60°C for 60 min. After that, 2 mL of 1 M H_2_SO_4_ in methanol was added and incubated at 60°C for 15 min. Following the two incubation steps, 2 mL of H_2_O and 2 mL of hexane were added to the tube, vortexed, then allowed the separation of two lipophilic and hydrophilic phases to take place. The hexane layer containing FAME was removed with a glass pipette and transferred into a gas chromatographic vial. GC was performed on an Agilent 7890B GC System, equipped with an FID and a capillary column Omegawax (30 m × 0.25 mm i.d., 0.25 µm film thickness). Injector and detector temperatures were maintained at 250°C and 260°C, respectively. The oven was set at 170°C for 1 min, and then it was increased by 1°C/min to 225°C. The carrier gas, helium, was used at a flow rate of 1.2 mL/min. The injection volume was 1 µL, with a split ratio of 10:1. Methyl esters of palmitic acid, stearic acid, oleic acid, linoleic acid, and linolenic acid were used as standards for fatty acid identification and quantification. The certified reference material F.A.M.E. Mix RM2 (Supelco, O71311AMP) was employed. Two biological replicates were technically repeated three times for the analysis.

### Statistics

All experiments were carried out in triplicate and repeated two times, and data on experimental microbial biomass and TFA contents were subjected to analysis of variance and Duncan’s multiple range test (*P* < 0.01) using SPSS 27.0 software. In the tables, alphabetic superscripts indicate the grouping ranges within which the varietal mean values are not significantly different.

## RESULTS

### Molecular identification of fungal species

**Fig 1:**
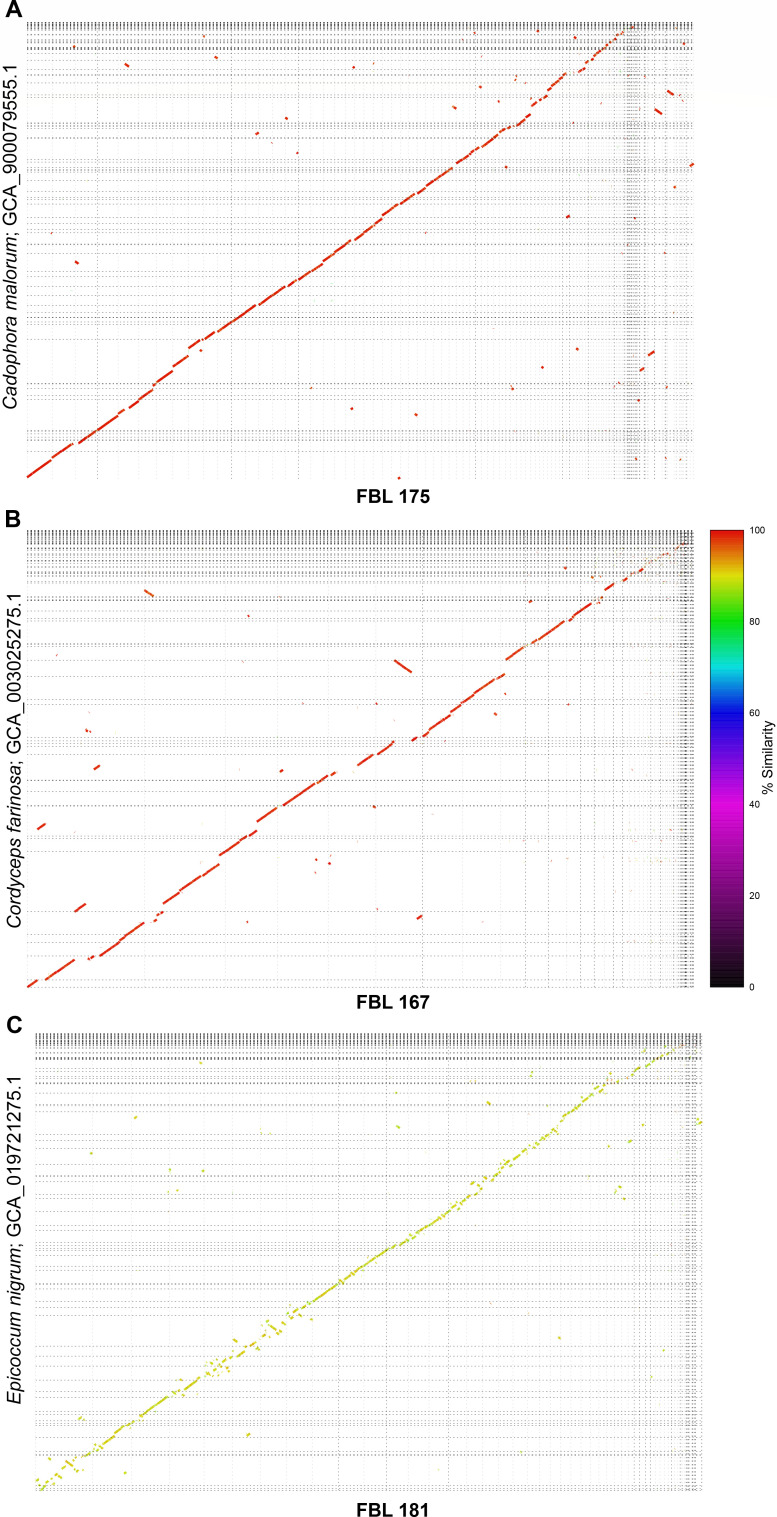
Dot plot of the fungal species sequenced for species identification. The dot plot represents the alignment with the best matching genome among all the analysis genomes present at RefSeq database at NCBI. (**A**) Alignment between FBL 175 and *Cadofora malorum* assembly GCA_900079555.1, (**B**) alignment between FBL 167 and *Cordyceps farinosa* assembly GCA_003025275, and (**C**) alignment between FBL 181 and *Epicoccum nigrum* GCA_019721275. Segments colors represent the level of similarity between the two genomes.

The strains FBL 167, FBL 175, and FBL 181 were characterized by Sanger sequencing of the internal transcribed spacer (ITS) region (Table S2). BLAST analysis of the three ITS regions showed that the sequence from the isolate FBL 167 matched the MN588141.1 representative for *Cordyceps farinosa* (syn. *P. farinosus*) clone TR-52-014; the sequence from the isolate FBL 175 matched *C. fastigiata* (syn. *Phialophora fastigiata*) strain F-30 (MF077223.1), while the sequence derived from isolate FBL 181 matched a fungal strain of *Agonomycetales* and shared 98.99% identity and 99% query coverage with *E. nigrum* isolate HG12 (KX099630.1). To further strengthen BLAST analysis performed by the ITS region, the genomes of the three isolates were sequenced by Oxford Nanopore Technologies sequencing. The genome size of isolate FBL 167 resulted in 37,275,427 bases in 15 contigs; the genome size of isolate FBL 175 was estimated at 46,261,765 bases in 91 contigs, while isolate FBL 181 was smaller, including 35,773,789 bases over 93 contigs. To assign a taxonomical species, each genome assembly was aligned to about 8,500 fungal genomes retrieved from the Assembly NCBI database. The alignment between the genome assembly of isolate FBL 175 showed a good identity with *C. malorum* strain M34 (GCA_900079555.1), with an identity of about 97.7%, suggesting that the identification via ITS is of a good quality (Fig. 1A). The genome of FBL 167 had the best alignment with the sequenced *Cordyceps farinosa* strain KACC 47486 (homotypic synonym *P. farinosus;* GCA_003025275.1), with an identity of about 97.9% (Fig. 1B), while the genome of isolate FBL 181 had the best match with *E. nigrum* genome (GCA_019721275.1) from strain P16 (GCA_019721275.1), with an identity of about 89.5% (Fig. 1C). Although the identity is quite low, this is the best match that we could get, confirming the ITS identification.

### Fungal growth evaluation

The effects of several carbon and nitrogen sources on biomass are shown in Table S3. For each fungus, the results showed differences in production, which depend on the substrate used for growth. Subsequent tests were conducted with substrates S10, S11, and S15. These substrates were chosen for the high production values, and S15 was also chosen to test the potential of agri-food processing wastes (milk whey and brewery wastes) as low-cost growth substrates for fungi (24).

Table 1 shows the production of fungal biomass in shaken flasks, expressed in grams of dry biomass per liter of culture medium, under different conditions of substrate and temperature. The results show that production of biomass in shaken flasks varies from ~20 to ~51 g/L of culture broth. In shaken flask cultivation, the yields vary from ~21 to ~38 g/L of culture broth for FBL 167, from ~28 to ~51 g/L of culture broth for FBL 175, and from ~20 to ~39 g/L for FBL 181. The maximum biomass production was always obtained at 25°C and 10°C on S15, except for FBL 181 in flask at 10°C.

**TABLE 1:** Effect of temperature and culture medium on the yield of biomass in flask^*a*^

Species	T (°C)	Flask (grams dry biomass per liter of culture medium [g/L])		
		S10	S11	S15
FBL 167	10	30.7 ± 3.6^b^	30.6 ± 2.6^b^	30.1 ± 3.1^b^
	25	29.2 ± 3.5^b^	20.9 ± 1.6^a^	29.7 ± 2.6^b^
	25 and 10	31.1 ± 3.1^b^	22.1 ± 2.9^a^	**37.6 ± 2.8** ^**c**^
FBL 175	10	37.1 ± 1.6^c^	28.6 ± 3.2^a^	39.3 ± 2.5^c^
	25	28.0 ± 2.5^a^	33.8 ± 3.1^b^	34.1 ± 1.9^b^
	25 and 10	27.8 ± 1.9^a^	35.1 ± 2.6^b^	**51.1 ± 3.8^d^**
FBL 181	10	**38.9 ± 1.9^d^**	33.8 ± 2.6^c^	35.9 ± 2.1^c,d^
	25	28.7 ± 3.6^b^	25.6 ± 1.9^a,b^	20.2 ± 3.8^a^
	25 and 10	34.9 ± 2.4^c,d^	26.3 ± 2.5^b^	29.0 ± 3.6^b^

^*a*^ The experiment was carried out maintaining the temperature for 10 days at 10°C (10); for 10 days at 25°C (25); for the first 6 days at 25°C, then lowered it to 10°C for the last 4 days (25 and 10). In bold, the highest values obtained for each fungus in flask are shown. Values for the same fungus that do not share the same alphabetic superscripts are significantly different according to Duncan’s multiple range tests (*P* < 0.01).

Scale-up from shaken flasks to the bioreactor is aimed at producing fungal biomass in large quantities and improving specific production. Therefore, the fungal culture was performed in a 5 L bioreactor, where several operational parameters such as pH, dissolved oxygen, mass mixing, etc., which are the key factors for the success of the fermentation, were controlled. Based upon shaken flasks results, i.e., PUFAs augment thanks to the 25° to 10°C “switch,” we grew the strains in the three media following the same protocol that cooled down to 10°C the fungal biomass after 5 days of growth at 25°C.

In the bioreactor, the best results in terms of mycelium biomass were achieved with the S15 substrate for all strains (obtaining from ~55 to ~59 g dry biomass per liter of culture medium) even in comparison with those obtained in shaken flasks (Table 2). Specifically, yields of 59.5, 56.2, and 54.7 g dry biomass per liter of culture medium for FBL 167, FBL 181, and FBL 175, respectively, were obtained, while production ranging from 32.2 to 37.5 and 31.3 to 33.2 was obtained for the substrates S10 and S11, respectively.

**TABLE 2:** Effect of temperature and culture medium on the yield of biomass in bioreactor^*a*^

Species	T (°C)	Biostat (grams dry biomass per liter of culture medium [g/L])		
		S10	S11	S15
FBL 167	25 and 10	32.0 ± 2.6^a^	33.2 ± 3.2^a^	**59.5 ± 3.6^b^**
FBL 175	25 and 10	39.3 ± 3.2^a^	37.0 ± 2.8^a^	**54.7 ± 2.9^b^**
FBL 181	25 and 10	37.5 ± 2.3^b^	31.3 ± 2.7^a^	**56.2 ± 2.5** ^**c**^

^*a*^ The experiment was carried out maintaining the temperature for the first 5 days at 25°C, then lowered it to 10°C for the last 3 days. In bold, the highest values obtained for each fungus in Biostat are shown. Values for the same fungus that do not share the same alphabetic superscripts are significantly different according to Duncan’s multiple range tests (*P* < 0.01).

### Determination of fatty acid content in fungi

**Fig 2:**
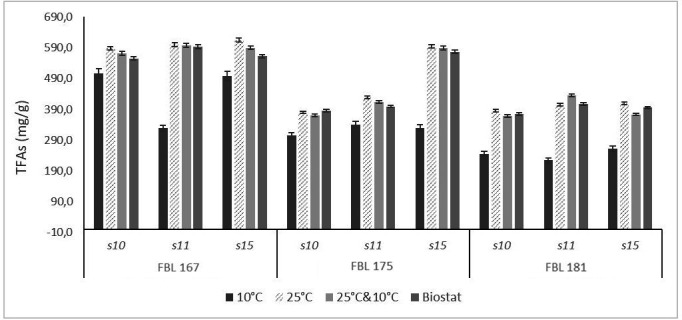
TFAs in FBL 167, FBL 175, and FBL 181 grown on different substrates (S10, S11, and S15) at two different temperature conditions: 10°C for 10 days; 25°C for 6 days, then lowered to 10°C for 4 days (25°C and 10°C); and 25°C for 5 days, then lowered to 10°C for 3 days (Biostat). “g” indicates mycelial dry weight. Results with standard error are means of two biological replicates, each technically repeated four times. Values for the same fungus and medium that do not share the same alphabetic superscripts are significantly different according to Duncan’s multiple range tests (*P* < 0.01) using SPSS software.

Here, we show the results of the GC analysis of FBL 167, FBL 175, and FBL 181 grown on S10, S11, and S15 at 10°C, 25°C, and 25°C and 10°C. As shown in Fig. 2, the total fatty acid (TFA) contents ranged between ~220 and ~600 mg/g mycelial dry weight (DW). TFA contents are lower at 10°C, and the values at 25°C and 10°C in the flasks were confirmed in the bench-scale bioreactor. At 25°C and 10°C and in the bioreactor, a higher TFA content in FBL 167 grown on all media and in FBL 175 grown on S15 was observed.

The highest percentages of PUFAs were observed with FBL 181 on S10 at 10°C (~77% of total fatty acids are PUFAs). The PUFAs in FBL 167 comprised between ~62% and ~66% of the TFAs. In FBL 175, PUFA amounts varied from a minimum of ~55% to a maximum of ~72%. The percentages of PUFAs in FBL 181 ranged between ~52% and ~77%. The highest concentration of PUFAs was observed with FBL 167 on S11 at 25 and 10°C (388.6 mg/g of mycelia dry weight). In FBL 175, we observed the maximum unsaturated fatty acid (UFA) value on S15 at 25 and 10°C (364.1 mg/g mycelial DW). In FBL 181, we observed the maximum UFA value on S11 at 25 and 10°C (346.9 mg/g mycelial DW) (Table 3). The highest values at 25°C and 10°C in the flasks were confirmed in the bench-scale bioreactor, except for FBL 181, which showed the maximum value on S15 instead of S11 (Table 4).

**TABLE 3:** Unsaturated fatty acid production in FBL 167, FBL 175, and FBL 181 grown in flask at different temperature and culture media^*a*^

Species	T (°C)	Unsaturated fatty acid production					
		S10		S11		S15	
		mg/g	%	mg/g	%	mg/g	%
FBL 167							
	10	332.3 ± 3.6^b^	65.6	216.3 ± 1.9^a^	65.8	311.8 ± 3.3^a^	62.5
	25	350.0 ± 3.0^b^	59.9	363.6 ± 3.0^b^	60.6	349.5 ± 3.5^b^	61.8
	25 and 10	362.7 ± 2.3^e^	63.4	**388.6** ± 3.1^c^	64.8	373.2 ± 3.5^c^	63.3
FBL 175							
	10	216.1 ± 1.8^a^	72.5	246.2 ± 2.6^a^	72.4	181.3 ± 1.3^a^	54.9
	25	249.8 ± 2.9^b^	62.7	229.8 ± 3.9^b^	67.5	351.6 ± 2.0^b^	59.0
	25 and 10	252.5 ± 2.4^b^	68.2	299.9 ± 2.7^c^	72.4	**364.1** ± 3.4^c^	61.8
FBL 181							
	10	167.7 ± 1.6^a^	76.7	158.8 ± 1.7^a^	69.2	136.3 ± 1.9^a^	52.0
	25	248.2 ± 1.5^b^	64.3	262.9 ± 3.4^b^	64.7	214.0 ± 2.4^b^	64.1
	25 and 10	257.4 ± 1.8^e^	70.0	**324.4** ± 3.0^c^	78.5	246.9 ± 2.7^c^	66.1

^*a*^ The experiment was carried out maintaining the temperature for 10 days at 10°C (10); for 10 days at 25°C (25); for the first 6 days at 25°C, then lowered it to 10°C for the last 4 days (25 and 10). Values are expressed as concentration on total mycelial dry weight and as a percentage with respect to TFAs. Values for the same fungus and medium that do not share the same alphabetic superscripts are significantly different according to Duncan’s multiple range tests (*P* < 0.01). In bold, the highest values obtained for each fungus in flask are shown.

**TABLE 4:** Unsaturated fatty acid composition in FBL 167, FBL 175, and FBL 181 grown in bioreactor on different culture media^*a*^

Species	Unsaturated fatty acid production					
	S10		S11		S15	
	mg/g	%	mg/g	%	mg/g	%
FBL 167	355.9 ± 2.5^a^	64.1	**389.5** ± 3.0^c^	65.7	365.9 ± 3.6^b^	65.0
FBL 175	307.4 ± 2.6^b^	79.7	282.5 ± 2.3^a^	70.7	**394.0** ± 3.2 ^c^	68.3
FBL 181	255.2 ± 2.5^a^	68.2	257.3 ± 2.0^a^	63.2	**261.3** ± 2.4 ^b^	66.8

^*a*^ Fungi grown on S10, S11, and S15 at a temperature of 25°C for 5 days and then cooled at 10°C for another 3 days (25°C and 10°C). Values are expressed as concentration on total mycelial dry weight and as a percentage with respect to TFAs. Values for the same fungus that do not share the same alphabetic superscripts are significantly different according to Duncan’s multiple range tests (*P* < 0.01). In bold, the highest values obtained for each fungus in Biostat are shown.

Tables 5 and 6 show the fatty acid qualitative-quantitative profile of the fungal strains: C16:0 palmitic (14%–36%), C18:0 stearic (6%–19%), C18:1 oleic (16%–48%), C18:2 linoleic (16%–44%), and C18:3 linolenic (0.2%–19%) acids. The calibration curve and full GC quantification data are provided in the Supplementary Material – GC quantification data file. These fatty acids were present in all the fungal strains analyzed. The most abundant fatty acids were palmitic, oleic, and linoleic acids, representing ~70–90% of the TFAs analyzed. In all the samples, the percentages of linolenic acid (18:3) were the highest at 10°C, while the lowest values were observed at 25°C, regardless of the substrate used. FBL 175 showed the highest concentration of linolenic acid under every growth condition tested in both flasks and bioreactor.

**TABLE 5:** Palmitic, stearic, oleic, linoleic, linolenic acid composition in Antarctic fungal species grown in flakes at different conditions and culture media^*a*^

Species	T(°C)	Substrate S10					Substrate S11					Substrate S15				
		C16:0	C18:0	C18:1	C18:2	C 18:3	C16:0	C18:0	C18:1	C18:2	C 18:3	C16:0	C18:0	C18:1	C18:2	C18:3
FBL 167	10	126.6 ± 3.8	47.6 ± 4.6	174.2 ± 1.5	145.9 ± 1.8	**12.2** ± 0.3^c^	76.6 ± 4.5	35.8 ± 0.4	90.7 ± 0.5	114.4 ± 1.1	11.2 ± 0.3^c^	106.2 ± 1.5	80.8 ± 0.5	170.6 ± 1.5	129.2 ± 1.5	12.0 ± 0.3^c^
	25	170.6 ± 1.5	67.6 ± 5.5	161.2 ± 1.8	186.5 ± 1.1	2.4 ± 0.1^a^	142.2 ± 2.0	70.8 ± 0.4	223.8 ± 2.2	159.0 ± 0.7	4.8 ± 0.1^a^	154,4 ± 0.9	92,9 ± 0.4	224.6 ± 2.1	121.6 ± 0.6	3.7 ± 0.1^a^
	25 and 10	159.0 ± 2.0	50.3 ± 0.5	192.8 ± 1.1	162.5 ± 0.9	7.4 ± 0.3^b^	144.5 ± 15	66.6 ± 4.7	215.9 ± 2.1	166.1 ± 0.7	6.6 ± 0.3^b^	138.4 ± 0.7	77.8 ± 0.3	232.3 ± 2.0	133.2 ± 1.4	7.7 ± 0.2^b^
FBL 175	10	61.4 ± 0.4	20.6 ± 0.2	82.6 ± 0.6	59.0 ± 0.6	**74.5** ± 0.6^c^	64.6 ± 0.5	29.2 ± 0.2	84.3 ± 0.9	106.8 ± 0.9	55.1 ± 0.8^c^	87.2 ± 0.3	61.8 ± 0.3	63.4 ± 0.4	54.5 ± 0.5	63.4 ± 0.4^c^
	25	82.5 ± 0.6	40.3 ± 0.7	82.9 ± 0.4	152.5 ± 1.1	14.4 ± 0.3^a^	111.5 ± 0.7	83.2 ± 0.5	98.6 ± 1.5	117.9 ± 2.1	13.3 ± 0.3^a^	143.2 ± 2.0	101.3 ± 0.3	164.5 ± 1.0	162.7 ± 0.7	24.4 ± 0.3^a^
	25 and 10	86.6 ± 0.4	31.1 ± 0.2	60.7 ± 0.5	141.8 ± 1.0	50.0 ± 0.3^b^	81.6 ± 0.5	32.7 ± 0.2	111.0 ± 1.4	139.6 ± 2.2	49.3 ± 0.3^b^	141.4 ± 1.2	83.7 ± 0.3	155.0 ± 1.9	151.4 ± 2.0	57.7 ± 0.5^b^
FBL 181	10	29.8 ± 0.4	21.2 ± 0.2	91.0 ± 0.5	70.4 ± 0.8	6.3 ± 0.3^c^	40.9 ± 0.4	29.8 ± 0.3	67.7 ± 0.5	84.9 ± 1.0	6.2 ± 0.2^c^	89.4 ± 0.8	36.5 ± 0.3	67.9 ± 0.8	61.6 ± 0.8	**6.8** ± 0.3^c^
	25	67.7 ± 0.5	52.0 ± 0.8	192.1 ± 2.2	87.8 ± 0.8	3.2 ± 0.3^a^	85.1 ± 1.0	57.9 ± 0.5	153. ± 2.4	106.9 ± 0.9	2.0 ± 0.1^a^	143.5 ± 1.0	52.9 ± 0.8	131.6 ± 1.3	81.6 ± 1.0	0.8 ± 0.1^a^
	25 and 10	57.7 ± 0.3	53.0 ± 0.6	136.1 ± 1.2	117.3 ± 1.0	4.0 ± 0.3^b^	72.7 ± 0.5	38.3 ± 0.2	184.6 ± 1.5	137.2 ± 1.2	2.6 ± 0.1^b^	91.2 ± 1.0	35.5 ± 0.3	130.4 ± 1.2	114.3 ± 1.2	2.2 ± 0.3^b^

^*a*^ Fungi grown on S10, S11, and S15 at 10°C for 10 days, at 25°C for 10 days, and at 25°C for 6 days, then lowered to 10°C for 4 days (25°C and 10°C). Values are expressed in mg/g of mycelial dry weight. Values for the same fungus and medium that do not share the same alphabetic superscripts are significantly different according to Duncan’s multiple range tests (*P* < 0.01). In bold, the highest percentages of linolenic acid (18:3) obtained for each fungus are shown.

**TABLE 6:** Palmitic, stearic, oleic, linoleic, linolenic acid composition in Antarctic fungal species grown in bioreactor at different condition and culture media^*a*^

Species	T(°C)	Substrate S10					Substrate S11					Substrate S15				
		C16:0	C18:0	C18:1	C18:2	C 18:3	C16:0	C18:0	C18:1	C18:2	C 18:3	C16:0	C18:0	C18:1	C18:2	C18:3
FBL 167	25 and 10	134.2 ± 1.1	65.3 ± 0.5	180.4 ± 1.3	159.3 ± 1.1	**16.2** ± 0.1^a^	118.2 ± 1.1	85.4 ± 0.8	209.0 ± 1.5	168.2 ± 1.4	12.3 ± 0.1^b^	112.5 ± 1.1	84.5 ± 0.7	214.8 ± 2.0	140.5 ± 1.3	10.6±0.3^c^
FBL 175	25 and 10	56.1 ± 0.4	22.4 ± 0.3	74.9 ± 0.3	168.5 ± 1.8	64.0 ± 0.5^a^	79.9 ± 0.3	37.0 ± 0.3	94.3 ± 0.7	137.9 ± 1.3	50.3 ± 0.3^b^	86.2 ± 0.3	96.8 ± 0.5	169.9 ± 1.3	158.2 ± 1.5	**65.8** ± 0.4^a^
FBL 181	25 and 10	54.8 ± 0.3	64.6 ± 0.8	124.6 ± 0.8	120.4 ± 1.4	**10.2** ± 0.3^a^	94.2 ± 0.7	55.9 ± 0.9	141.8 ± 1.1	112.3 ± 0.8	3.2 ± 0.1^b^	80.3 ± 0.8	53.9 ± 0.4	137.2 ± 1.3	117.3 ± 0.9	6.8 ± 0.2^c^

^*a*^ Fungi grown on S10, S11, and S15 at temperature of 25°C for 5 days and then cooled at 10°C for the other 3 days (25°C and 10°C). Values are expressed in mg/g of mycelial dry weight. In bold, the highest percentages of linolenic acid (18:3) obtained for each fungus are shown. Values for the same fungus that do not share the same alphabetic superscripts are significantly different according to Duncan’s multiple range tests (*P* < 0.01).

Oleic, linoleic, and linolenic acids are present in all the strains and in every culture condition in high amounts, and considering the biomass obtained with S15, i.e., with a medium amended with agri-food wastes, we can obtain, for instance, 21 g/L of UFAs and 3.5 g/L of linolenic acid after 10 days of growth in the bioreactor.

## DISCUSSION

Aware that microorganisms adapted to live in permanently cold environments represent a largely unexplored bioresource for potential biotechnological applications, we aim to characterize phenotypical traits in the production of PUFAs in Antarctic fungi to provide nature-based solutions for sustainable PUFA production. The idea of identifying oleaginous species among those adapted to live in habitats with temperatures close to zero was plausible, since the essential role of highly PUFAs stored in membrane phospholipids to maintain membrane functionalities in permanently cold environments is known (25). Changes in lipid metabolism are the major responses through which acclimatization and adaptation to cold can occur in microorganisms. The function of the lipid membranes is compromised at temperatures near freezing, which is linked to maintaining the appropriate physiological state (15, 26).

Different microorganisms, bacteria, yeasts, filamentous fungi, and microalgae (27, 28), among others, can synthesize long-chain PUFAs. Such microorganisms are at least partly easily scaled up for cultivation purposes and can grow on abundant and low-cost cultivation media (29). These alone can reduce enormously the cost of producing and, hence, extracting oils from these microorganisms. More specifically, fungi easily modify their lipid pattern because of their growing substrates (30). Therefore, it is important to identify growth parameters to optimize polyunsaturated fatty acid production (31, 32). While *C. farinosa* has not been deeply profiled for fatty acids in the Antarctic environment, it appears in broader lipid biomarker studies of fungal decomposers. *C. farinosa* was considered for the analysis of sterols and fatty acids in natural and cultured conditions. This offers some insight into its lipid biosynthesis under cold-environment growth conditions (33, 34). *C. malorum*, a species previously isolated from Antarctic soils, is known for its ability to cause soft-rot decay by degrading wood (35) and the synthesis of cyclic heptapeptides with metal-binding properties. These peptides, due to their ring structure, can exhibit enhanced stability and resistance against proteolytic degradation in cold-related stress (36). Recent investigations have confirmed the presence of *E. nigrum* in the Antarctic Peninsula through complementary approaches, including metabarcoding analyses that revealed its occurrence within the fungal communities associated with glacial environments (37), as well as culture-based isolation studies in which *E. nigrum* was consistently recovered from all examined moss and algal samples (38). A study has already characterized some fatty acids of *E. nigrum* strains isolated from Patagonian fiords. The predominant fatty acids identified were C16:0, C16:1, C18:0, C18:1, and C18:2, collectively accounting for approximately 88% of the total fatty acid content, but no significant differences were detected in the relative proportions of saturated and unsaturated fatty acids between *E. nigrum* cultures grown at different temperatures, 6°C and 25°C. Conversely, temperature had a pronounced impact on sterol production (39).

To simplify and render economically sustainable PUFA production from these fungi, we identify waste materials that can support their growth and, furthermore, select those that can support the synthesis of PUFAs at low temperatures. High yields of mycelial dry matter, ranging from 5.9 to 12.9 g/L, were achieved in many of the substrates tested in preliminary screening (Table S1) when compared with those obtained in PD broth (substrate S1). Liu et al. and Mascarin et al. (40, 41), culturing *Isaria farinosa* (syn. *C. farinosa*) in shake flasks with PDB at 25°C, reported yields of 5.8 and 4.3 g/L, respectively. While Salehi et al. (42) for *E. nigrum* achieved values of biomass between 3 and 12 g/L on Murashige and Skoog medium. Vadivelan et al. (43) for *Mortierella alpina* achieved values of biomass between 8.4 and 11.8  g/L on starch-yeast extract medium and 16.7  g/L mycelial DW with the addition of linseed oil and garden cress oil (44). The highest yields obtained with our set of media (except S12) could be due to the high concentration of carbon sources. This claim is supported by the findings observed for other fungal species in several studies. Zhu et al. (45) in shake flasks obtained 31.2 g/L of dry biomass culturing *M. alpina* on an inexpensive medium composed of maize starch hydrolysate (with glucose concentration of 100 g/L) and different nitrogen sources. Kim et al. (46) reported that in shake-flask cultures, the mycelial growth of *Paecilomyces sinclairii* was proportional to the increase of sucrose concentration within the range from 10 to 60 g of sugar/L. In the bioreactor, the best results in terms of mycelium biomass were achieved with the S15 substrate for all strains. In particular, the highest value of 59.5 g/L was reached for *P. farinosus*. Overall, in this study, we obtained higher biomass yields using the bioreactor rather than the shake flasks. In fact, by controlling the aeration and agitation rates, it can be possible to increase oxygen supply, to improve mixing, and to enhance the mass transfer (47). In a recent study, Supramani et al. (48) showed that the biomass yield of *Ganoderma lucidum* was 12.31 g/L in the shake flasks and 53.96 g/L in an innovative bioreactor. Also, these authors concluded that the higher bioreactor yield was achieved through improvement of oxygenation in the medium during the fungal growth (48). This result was also supported by the study of Rodrigues et al. (49), for the biomass production of a recombinant *Pichia pastoris*.

Parallel to the optimization/tuning of the growth substrate and culture condition (shake flasks and bioreactor) to enhance the capability to yield more biomass compared to traditional, expensive substrates, we checked the abilities that these strains isolated in Antarctica possess regarding the synthesis of PUFAs. With this further purpose in mind, we have selected three modalities of growing these isolates, focusing on the role of temperature (i.e., 10°C, 25°C, 25 and 10°C).

The condition 25°C and 10°C (in flask and bioreactor) allows the obtainment of a higher biomass (except for FBL 181) and TFA content than the condition at 10°C observing a total fatty acid content up to about 600 mg/g (DW). Although under different conditions, Ho and Chen (50) obtained a total fatty acid content in *M. alpina* up to about 400 mg/g (DW). PUFA amounts were higher than those of saturated fatty acids with percentages ranging between 52 and 77%. Unlike what has been observed so far, the percentages of linolenic acid (18:3), a precursor of complex lipids of great interest for human health (51), were higher when the fungi grew at 10°C compared to growth at 25°C and 10°C (while the lowest values were obtained when the fungi grew at 25°C). Maggi et al. (52) observed that in the cold experimental condition (8°C), a higher concentration of linolenic acid with respect to growth at 25°C was observed for different strains including *C. fastigiata*, a species belonging to the same genus as *C. malorum*. Jang et al. (53) observed that a high degree of unsaturation was found at a low-temperature incubation. Xian et al. (54) also showed that the proportion of linolenic acid increased with a decrease in cultivation temperature, reaching a maximum yield at 5°C. A possible explanation for these observations derives from the fact that at lower temperatures, more oxygen is dissolved in water and, thus, more oxygen is available for the oxygen-dependent desaturase enzymes (55, 56).

In our results, palmitic (16:0), stearic (18:0), oleic (18:1), linoleic (18:2), and linolenic (18:3) acids were present in every fungal strain, confirming the literature data (57). These results overlap with those of Foppen et al. (58) for *E. nigrum*, by Stahl and Klug (59), who determined that these fatty acids were the most common and abundant after analyzing 100 strains of filamentous fungi, including Mucoromycetes, Ascomycetes, Basidiomycetes, and Agonomycetales (formerly *Micelia sterilia*). A recent study investigated the lipid production in cultivable filamentous fungi isolated from Antarctic soils (60). The FA profile of such fungi (at least 18 different species were examined) grown at an intermediate low temperature (10°C) was comparable to what was obtained in our study. Notably, oleic and linoleic acids were the most represented in each of the tested species. This is not dissimilar to what we can infer from our results, considering that the real switch to a higher quantity of linolenic acid was obtained at 4°C (at least in comparison with oleic acid).

Fungi are formidable, continuously transforming and adapting organisms. The latter news on climate change indicates these organisms as the most capable of adapting to timely changing climate conditions, posing, indeed, a threat to crop production (61). Nevertheless, these capacities represent an opportunity to be exploited. For instance, fungi can serve as bioremediators (62), biopesticides (63), biofactories (64), among others. They can sense the environment and quite instantly adapt their growth to resist stress and use new substrates for nutrients (65). In this process, extreme organisms like those adapting to extreme climate conditions (e.g., Antarctica) can serve as inspiration for nature-based solutions to present and future problems.

The values of PUFAs obtained in this study from fungi isolated in Antarctica are particularly noteworthy. Total fatty acid contents reached up to 600 mg/g DW, surpassing many previously reported values for oleaginous fungi such as *Mortierella alpina* (50), which typically produces around 400 mg/g DW. This high yield suggests strong adaptation to cold environments that necessitates increased membrane fluidity, achievable through higher PUFA content. Proportions of PUFAs between 52% and 77% of total fatty acids further confirm a metabolic preference toward unsaturation at low temperatures. Importantly, the increase of linolenic acid (C18:3) at 10°C highlights specific desaturation pathway activation likely mediated by enhanced oxygen solubility and enzyme activity at lower temperatures (52–56). These biochemical adaptations are consistent with membrane homeostasis mechanisms well documented in such psychrophilic microorganisms (15, 26).

From an applied perspective, the characterization of phenotypical traits of these fungal strains suggests that combination of high biomass production—especially in bioreactor conditions—and substantial PUFA content, combined with the use of inexpensive agri-food waste substrates, can be an excellent combination for more sustainable lipid production. The innovation of our study consists of the characterization of the phenotypical traits of PUFA production by Antarctic fungal strains in different cultural conditions, which suggests a new sustainable PUFA production by microorganisms from extreme environments. PUFAs can be used for several applications: biofuels (66) (alternative to algae), drug delivery (67), antimicrobials (68), biodegradable polymers (69), and high-value nutraceuticals (70). In this context, our results suggest a new potential way to produce PUFAs by microorganisms from extreme environments in a more sustainable way, using agri-food processing wastes.

## Data Availability

All data produced in this paper are deposited at the NCBI database under the project number PRJNA1298289 (Biosample accessions: SAMN50284729: FBL175; SAMN50263805: FBL181; SAMN50284730: FBL167).

## SUPPLEMENTAL MATERIAL

Data Set S1 spectrum.01372-25-s0001.xlsx Palmitic, stearic, oleic, linoleic, and linolenic acid composition in Antarctic fungal species.

Data Set S2 spectrum.01372-25-s0002.xlsx Total FA.

Supplemental tables spectrum.01372-25-s0003.docx Tables S1 to S3.

ASM does not own the copyrights to Supplemental Material that may be linked to, or accessed through, an article. The authors have granted ASM a non-exclusive, world-wide license to publish the Supplemental Material files. Please contact the corresponding author directly for reuse.
