# Supplementary material for: Antarctic fungi: a bio-source alternative to produce polyunsaturated fatty acids (PUFAs)
Source: Microbiol Spectr. 2026 Jan 13;14(2):e01372-25. doi: 10.1128/spectrum.01372-25 (PMC12889145; doi:10.1128/spectrum.01372-25)
Supplement: Supplemental tables — Tables S1 to S3. [file spectrum.01372-25-s0003.docx]

SUPPLEMENTAL MATERIAL

ANTARCTIC FUNGI: A BIO-SOURCE ALTERNATIVE TO PRODUCE POLYUNSATURATED FATTY ACIDS (PUFAs)

Patrizia De Rossi^*^, Alfredo Ambrico, Antonella Del Fiore, Mario Trupo, Luciano Blasi, Marzia Beccaccioli, Luigi Faino, Andrea Ceci, Oriana Maggi, Anna Maria Persiani, Massimo Reverberi

To whom correspondence should be addressed: [patrizia.derossi@enea.it](mailto:patrizia.derossi@enea.it)

Running Title: ANTARCTIC FUNGI ALTERNATIVE TO PRODUCE PUFAs

Keywords: microfungi, polyunsaturated fatty acid, biomass, PUFAs, Antarctic fungi, linolenic fatty acid, bio-sources

**SUPPLEMENTAL DATA**

**TABLE S1.** Composition of the media used in this study.

| Substrate | Composition | | | | | | |
| --- | --- | --- | --- | --- | --- | --- | --- |
| S1 | PDB | 24 g/L |  |  |  | |  |
| S5 | Glycerol | 100 g/L | KNO_3_ | 10 g/L | | Yeast extract | 5 g/L |
| S6 | Glucose | 100 g/L | KNO_3_ | 10 g/L | | Yeast extract | 5 g/L |
| S7 | Glucose | 100 g/L | KNO_3_ | 10 g/L | | Corn steep liquor | 5 g/L |
| S10 | Sucrose | 100 g/L | KNO_3_ | 10 g/L | | Yeast extract | 5 g/L |
| S11 | Maltose | 100 g/L | KNO_3_ | 10 g/L | | Yeast extract | 5 g/L |
| S12 | Cellulose from paper | 100 g/L | KNO_3_ | 10 g/L | | Yeast extract | 5 g/L |
| S13 | Potato extract | 100 g/L | KNO_3_ | 10 g/L | | Yeast extract | 5 g/L |
| S15 | Sucrose | 60 g/L of Mw* | KNO_3_ | 10 g/L of Mw* | | Brewery wastes | 5 g/L of Mw* |

*Milk whey

**TABLE S2.** BLAST analysis of ITS regions from the isolate FBL 167, FBL 175 and FBL 181.

| No.  FBL culture collection | Cod. | Specie  Phenothype identification | Specie  Identification with ITS | % similarity  GenBank (NCBI) | No. GenBank  (NCBI) |
| --- | --- | --- | --- | --- | --- |
| 167 | **4** | *Paecilomyces farinosus* | *Paecilomyces farinosus* | 99% *P. farinosus* | MN588141.1 |
| 175 | **8** | *Phialophora fastigiata* | *Phialophora sp.* | 100%*Phialophora fastigiata* | MF077223.1 |
| 181 | **10** | *Agonomycetales* | *Epicoccum nigrum* | 99% *Epicoccum nigrum* | KX099630.1 |

>ITS *Paecilomyces farinosus*

ACTCCCAAACCCTTTTGTGATCATACCTATCGTTGCTTCGGCGGACTCGCCCCAGCGTCCGGCCGGCCCCGCGCCGGCCGCGGCCTGGATCCAGGCGGCCGCCGGAGACCCCCAAACTCTGTATTCTCAGTATCTTCTGAATCCGCCGCAAGGCAAAACAAATGAATCAAAACTTTCAAGAACGGATCTCTTGGTTCTGGCATCGATGAAGAACGCAGCGAAATGCGATAAGTAATGTGAATTGCAGAATTCAGTGAATCATCGAATCTTTGAACGCACATTGCGCCCGCCAGCATTCTGGCGGGCATGCCTGTTCGAGCGTCATTTCAACCCTCGACTTCCCTTTGGGGAAATCGGCGTTGGGGACCGGCCGTATACCGCCGGCCCCGAAATGAAGTGGCGGCCCGTCCGCGGCGACCTCTGCGTAGTAATCCAACTCGCACCGGAACCCCGACGTGGCCACGCCGTAAAACCCCCGACTTCTGAACGTTGACCTCGAATCAGGTAGGA ATACCCGCTG AACTTAAGCA TATCAATAA

>ITS *Cadophora malorum*

GTGAACCTGCGGAAGGATCATTACTAGAGCAAAGGATAGGCAGCGCCCCACCGAAGCTTGCTTCGTGGGGTGTCGAGCCGTCGACCCTCTCGGAGAAGGTCGGTCCTGAACTCCACCCTTGAATAAATTACCTTTGTTGCTTTGGCGGGCCGCCTCGCGCCAGCGGCTTCGGCTGTTGAGTGCCCGCCAGAGGACCACAACTCTTGTTTTTAGTGATGTCTGAGTACTATATAATAGTTAAAACTTTCAACAACGGATCTCTTGGTTCTGGCATCGATGAAGAACGCAGCGAAATGCGATAAGTAATGTGAATTGCAGAATTCAGTGAATCATCGAATCTTTGAACGCACATTGCGCCCTCTGGTATTCCGGGGGGCATGCCTGTTCGAGCGTCATTATAACCACTCAAGCTCTCGCTTGGTATTGGGGTTCGCGGTTCCGCGGCCCCTAAAATCAGTGGCGGTGCCTGTCGGCTCTACGCGTAGTAATACTCCTCGCGTCTGGGTCCGGTAGGTCTACTTGCCAGCAACCCCCAATTTTTACAGGTtGACCTCGGATC

>ITS *Epicoccum nigrum*

GACCTCGGAAGGATCATTACATAAAAGGATACCCTCACCGGTATACCCCACCCGTGTCTATCTACTCTTGTTGCTTTGGCAGGCCGTGGTCTCCCACTGTGGGCTTTGCCTGCATGTGCCTGCCAGAGGACCAAACTCTGAATTTTAGTGATGTCTGAGTACTATATAATAGTTAAAACTTTCAACAACGGATCTCTTGGTTCTGGCATCGATGAAGAACGCAGCGAAATGCGATAAGTAATGTGAATTGCAGAATTCAGTGAATCATCGAATCTTTGAACGCACATTGCGCCCTGTGGTATTCCGCAGGGCATGCCTGTTCGAGCGTCATTATAACCACTCAAGCCTCGCTTGGTGTTGGGGCTCGCAATCTTGCGGCCTCTAAAATCAGTGGCGGTGCCAGTAGGCTCTGAGCGTAGTACATCTCCTCGCTATAGAGTCCTATCGGTCCCCTGCCAAAACCCCCATATTTTTATAGGTGACCTC

**TABLE S3.** Fungal dry biomass produced at a temperature of 25 °C.

| Media | Dry biomass g/L | | | |
| --- | --- | --- | --- | --- |
|  | | *Paecilomyces farinosus* FBL 167 | *Cadophora malorum*  FBL 175 | *Epicoccum nigrum*  FBL 181 |
| S1 | | 5.9±3.8^a^ | 12.9±2.2^b^ | 6.0±2.9^a^ |
| S5 | | 26.8±2.5^c^ | 27.0±1.9^c^ | 14.2±2.2^b^ |
| S6 | | 23.5±1.5^c^ | 26.3±1.5^c^ | 28.0±3.2^d^ |
| S7 | | 26.8±3.9^c^ | 27.7±1.8^c^ | 28.2±1.2^d^ |
| S10 | | **29.2**±3.6^d^ | 28.0±2.2^c^ | **28.7**±3.1^d^ |
| S11 | | 20.9±3.9^b^ | **33.8**±1.9^d^ | 25.6±2.8^d^ |
| S12 | | 0.2±2.5^a^ | 0.0±2.7^a^ | 0.1±3.0^a^ |
| S13  S15 | | 16.8±1.7^b^  **29.7**±1.2^d^ | 5.7±3.6^a^  **34.1**±1.9^d^ | 11.6±2.7^b^  20.2±2.5^c^ |

In bold, the highest values obtained for each fungus are shown. Values for the same fungus that do not share the same alphabetic superscripts are significantly different in according to Duncan’s multiple range tests (*p* < 0.01).
